# Validation of the Seegene RV15 multiplex PCR for the detection of influenza A subtypes and influenza B lineages during national influenza surveillance in hospitalized adults

**DOI:** 10.1099/jmm.0.001032

**Published:** 2019-07-02

**Authors:** J. J. LeBlanc, M. ElSherif, S. Mulpuru, M. Warhuus, A. Ambrose, M. Andrew, G. Boivin, W. Bowie, A. Chit, G. Dos Santos, K. Green, S. A. Halperin, T. F. Hatchette, B. Ibarguchi, J. Johnstone, K. Katz, J. M. Langley, P. Lagacé-Wiens, M. Loeb, A. Lund, D. MacKinnon-Cameron, A. McCarthy, J. E. McElhaney, A. McGeer, A. Poirier, J. Powis, D. Richardson, M. Semret, V. Shinde, D. Smyth, S. Trottier, L. Valiquette, D. Webster, L. Ye, S. A. McNeil

**Affiliations:** ^1^​ Canadian Center for Vaccinology, Dalhousie University, IWK Health Centre, and Nova Scotia Health Authority, Halifax, NS, Canada; ^2^​ Ottawa Hospital Research Institute, University of Ottawa, Ottawa, ON, Canada; ^3^​ Centre Hospitalier Universitaire de Québec, QC, Canada; ^4^​ University of British Columbia, Vancouver, BC, Canada; ^5^​ Sanofi Pasteur, Swiftwater, PA, USA; ^6^​ Leslie Dan Faculty of Pharmacy, University of Toronto, Toronto, ON, Canada; ^7^​ Business & Decision Life Sciences (on behalf of GSK), Bruxelles, Belgium; ^8^​ Mount Sinai Hospital, Toronto, ON, Canada; ^9^​ GSK, Mississauga, ON, Canada; ^10^​ Public Health Ontario and University of Toronto, Toronto, ON, Canada; ^11^​ North York General Hospital, Toronto, ON, Canada; ^12^​ St Boniface Hospital, Winnipeg, MB, Canada; ^13^​ Ottawa Hospital General, Ottawa, Ontario, Canada; ^14^​ Health Sciences North Research Institute, Sudbury, ON, Canada; ^15^​ Centre Intégré Universitaire de Santé et Services Sociaux, Quebec, QC, Canada; ^16^​ Toronto East General Hospital, Toronto, ON, Canada; ^17^​ William Osler Health System, Brampton, ON, Canada; ^18^​ McGill University, Montreal, QC, Canada; ^19^​ GSK, King of Prussia, PA, USA; ^20^​ The Moncton Hospital, Moncton, NB, Canada; ^21^​ Université de Sherbrooke, Sherbrooke, QC, Canada; ^22^​ Horizon Health, Saint John, NB, Canada; ^†^​ Present address: GSK, Wavre, Belgium.; ^‡^​ Present address: Bayer, Inc., Mississauga, Ontario, Canada.; ^§^​ Present address: Novavax Vaccines, Washington, DC, USA

**Keywords:** influenza, multiplex PCR, subtype, lineage, validation

## Abstract

**Background:**

The Serious Outcomes Surveillance Network of the Canadian Immunization Research Network (CIRN SOS) has been performing active influenza surveillance since 2009 (ClinicalTrials.gov identifier: NCT01517191). Influenza A and B viruses are identified and characterized using real-time reverse-transcriptase polymerase chain reaction (RT-PCR), and multiplex testing has been performed on a subset of patients to identify other respiratory virus aetiologies. Since both methods can identify influenza A and B, a direct comparison was performed.

**Methods:**

Validated real-time RT-PCRs from the World Health Organization (WHO) to identify influenza A and B viruses, characterize influenza A viruses into the H1N1 or H3N2 subtypes and describe influenza B viruses belonging to the Yamagata or Victoria lineages. In a subset of patients, the Seeplex RV15 One-Step ACE Detection assay (RV15) kit was also used for the detection of other respiratory viruses.

**Results:**

In total, 1111 nasopharyngeal swabs were tested by RV15 and real-time RT-PCRs for influenza A and B identification and characterization. For influenza A, RV15 showed 98.0 % sensitivity, 100 % specificity and 99.7 % accuracy. The performance characteristics of RV15 were similar for influenza A subtypes H1N1 and H3N2. For influenza B, RV15 had 99.2 % sensitivity, 100 % specificity and 99.8 % accuracy, with similar assay performance being shown for both the Yamagata and Victoria lineages.

**Conclusions:**

Overall, the detection of circulating subtypes of influenza A and lineages of influenza B by RV15 was similar to detection by real-time RT-PCR. Multiplex testing with RV15 allows for a more comprehensive respiratory virus surveillance in hospitalized adults, without significantly compromising the reliability of influenza A or B virus detection.

## Introduction

Influenza virus infection is a leading infectious cause of morbidity and mortality in developed countries, and is of considerable public health concern [1–7]. Many vaccine formulations have been developed to reduce the burden of influenza illness, but the continued evolution of the viruses through antigenic drift requires that the vaccines be reformulated each year [8–10]. Monitoring the epidemiology and burden associated with circulating influenza viruses is important to make informed recommendations on vaccine use [8–10], and the currently circulating strains include influenza A virus subtypes H1N1 and H3N2 and the Yamagata and Victoria lineages of influenza B [11].

Since 2009, the Serious Outcomes Surveillance Network of the Canadian Immunization Research Network (CIRN SOS) has been conducting active surveillance for acute respiratory illness in hospitalized adults to monitor the burden of influenza illness and assess the effectiveness of seasonal influenza vaccines against laboratory-confirmed influenza [1–7]. The CIRN SOS Network comprises 15 to 45 acute care (depending on the year) hospitals across Canada, and influenza testing is performed using real-time reverse-transcriptase polymerase chain reaction (RT-PCR) methods derived from the World Health Organization (WHO) [12–14]. These methods allow the identification of influenza A and B, the discrimination of influenza A viruses into H1N1 and H3N2 subtypes, and the characterization of influenza B viruses into Yamagata or Victoria lineages [12–14]. These viruses were all circulating in Canada at the time of this study.

While WHO-based real-time RT-PCRs methods are often viewed as the reference standard for influenza virus detection, diagnostic laboratories and surveillance studies often test for other viral aetiologies of respiratory illness [15–28]. To avoid the high cost and labour associated with individual virus detection, multiplex RT-PCR technologies have been developed and are now commercially available [28]. Multiplex RT-PCR can detect influenza A and B, as well as non-influenza respiratory viruses (NIRVs), such as respiratory syncytial virus (RSV), coronaviruses, rhinoviruses, human metapneumovirus (hMPV), human parainfluenza viruses, adenovirus and enteroviruses [11, 15–28]. While the focus often lies on influenza, NIRVs can also be a significant causes of morbidity and mortality [29–36]. NIRVs can also co-circulate with influenza [11, 29], and can mirror the clinical presentations of influenza, or other viral or bacterial respiratory tract infections [30–36]. From a diagnostic perspective, rapid identification of viral respiratory viruses has potential benefits, such as a decrease in antibiotic prescriptions, a decrease in laboratory investigations and more judicious use of oseltamivir, and allows for the implementation of infection control practices, such as patient cohorting [31, 37, 38]. From a surveillance perspective, understanding the epidemiology of NIRVs can inform guidelines for patient management [9–11, 38] and help guide the development of new vaccines or therapeutics [31]. This study focuses on molecular detection of influenza viruses for the purpose of surveillance.

The CIRN SOS Network has performed testing for NIRVs on a subset of nasopharyngeal swabs collected for influenza surveillance. NIRV testing was performed using a Health Canada approved test, the Seeplex RV15 One-step ACE Detection assay (Seegene, Inc., Seoul, Republic of Korea) (RV15). This conventional multiplex RT-PCR uses dual-priming oligonucleotide (DPO) technology [39, 40] for the detection of influenza A and B, as well as 13 other respiratory viruses. The performance of RV15 has previously been compared against cell culture, direct immunofluorescence, real-time RT-PCR and other multiplex RT-PCRs for respiratory viruses [16–25, 36, 41]; however, to the best of our knowledge, the performance of RV15 for the detection of influenza A and B has yet to be compared against that of the WHO real-time RT-PCR reference methods. Given that RV15 showed variable performance for other viruses compared to other molecular methods [16, 17, 19, 36], an assessment of RV15 against the WHO reference methods for influenza A and B is well justified. Both RV15 and the WHO real-time RT-PCRs were used by the CIRN SOS Network, allowing a direct comparison of these methods.

## Methods

### Ethics

This study was approved by the research ethics boards (REBs) at each participating hospital (ClinicalTrials.gov identifier: NCT01517191). These included the William Osler Health System Research Ethics Board (Brampton, ON), the University of Edmonton Health Research Ethics Board (Edmonton, AB), the Capital Health Research Ethics Board (Halifax, NS), the Hamilton Health Sciences/McMaster Health Sciences Research Ethics Board (Hamilton, ON), the Horizon Health Network Research Ethics Board (Moncton, NB), the BMD Research Ethics Board MUHC – Montreal General Hospital (Montreal, QC), the Ottawa Health Science Network Research Ethics Board (Ottawa, ON), le Comité d'éthique de la recherche du CHU de Québec (Québec, QC), the Horizon Health Network Research Ethics Board (Saint John, NB), le Comité d'éthique de la recherche sur l’humain du Centre hospitalier de Sherbrooke (Sherbrooke, QC), the Health Sciences North Research Ethics Board (Sudbury, ON), the Mount Sinai Research Ethics Board (Toronto, ON), the North York General Research Ethics Board (Toronto, ON), the Toronto East General Hospital Research Ethics Board (Toronto, ON), le Comité d'éthique de la recherche de Trois-Rivières-Centre hospitalier affilié universitaire regional (Trois-Rivières, QC), the University of British Columbia Clinical Research Ethics Board (Vancouver, BC) and the University of Manitoba Health Research Ethics Board (Winnipeg, MB).

### Active surveillance by the CIRN SOS Network

Active surveillance for influenza was performed across up to 45 hospitals in 5 Canadian provinces over consecutive influenza seasons starting in 2009, but the specimens from this study were collected between November 2011 and May 2013. On a daily basis, dedicated SOS Network surveillance monitors reviewed all adult admissions (aged ≥16 years) to identify patients with an acute respiratory illness, and patient demographics and outcomes were collected. Patient demographics and outcomes have been the subject of several CIRN SOS Network publications [1–7].

### Specimen collection and processing

Within 7 days of onset of illness, consenting patients were enrolled and tested for influenza viruses A and B from NP swabs collected in universal transport media (UTM) (Copan Diagnostics). All swabs were divided into aliquots and archived at −80 °C for batch shipment on dry ice to the CIRN SOS Reference Laboratory at the Canadian Center for Vaccinology (CCfV) (Halifax, NS).

### Real-time RT-PCR

The CIRN SOS Reference Laboratory retested all specimens from each CIRN site using validated real-time RT-PCR methods [12–14]. Total nucleic acids (TNAs) were extracted from 140 µl of NP swab material using a MagNaPure LC 2.0 instrument (Roche Diagnostics). The TNAs were eluted into 60 µl, and 5 µl served as a template for each 25 µl real-time RT-PCR reaction consisting of 0.5 µl SuperScript III RT/Platinum *Taq* Mix PCR enzyme mix (Invitrogen, Carlsbad, CA, USA), 1×PCR Master Mix, 0.8 µM primers and 0.2 µM probes (Table S1, available in the online version of this article). Initially, influenza strains were identified as A or B with a duplex real-time RT-PCR using primers targeting the matrix genes for each influenza type (Table S1). Subsequently, TNA that was positive for influenza A was subjected to a real-time RT-PCR subtyping assay targeting the haemagglutinin (HA) genes specific to either H1 or H3. TNA that was positive for influenza B was subjected to real-time RT-PCR characterization into Victoria or Yamagata lineages. Amplifications of all real-time RT-PCR assays were performed on an Applied Biosystems 7500 Fast Instrument (Life Technologies), under the following conditions: reverse transcription at 50 °C for 30 min; activation of the *Taq* DNA polymerase at 95 °C for 2 min; and 45 cycles of 95 °C for 15 s (denaturation) and 55 °C for 30 s (combined annealing and extension). Threshold cycle (*C*_t_) values were provided by the manufacturer’s software, and the *C*_t_ cutoff for positivity was determined using previously validated values at defined thresholds (Table S2).

### RV15 respiratory virus multiplex PCR

Following the same TNA extraction method as described for real-time RT-PCR analyses, TNA was extracted from a separate aliquot of NP swab material and 10 µl of TNA was used as a template for RV15 reactions, as recommended in the manufacturer’s instructions. Amplification was performed in a 96-well plate in a C1000 Touch Thermocycler (BioRad Laboratories Ltd, Mississauga, ON, Canada). Amplicons were resolved using 1.2 % (w/v) agarose gel electrophoresis with staining using 1.0 µg ml^−1^ ethidium bromide (final concentration), and visualized on a GelDoc XR+instrument with ImageLab software (version 5.1) (BioRad Laboratories).

### Statistical analyses

Influenza A and B results from the RV15 or real-time RT-PCR assays were classified as positive or negative, and compared to a composite reference standard where concordant results between two of three methods were considered a true positive or negative result. Discrepant analyses were performed using real-time RT-PCR and/or sequencing by the National Microbiology Laboratory (Winnipeg, MB, Canada). Statistical Analysis Software (SAS) version 9.4 (SAS Institute, Cary, NC, USA) was used to assess significant differences between methods using 2×2 contingency tables and McNemar’s chi square test. A *P* value ≤0.05 was considered statistically significant. Sensitivity, specificity, accuracy, misclassification rates and kappa statistics were reported with 95 % confidence intervals (CIs).

### Cloning of the matrix gene targets of influenza A and B and viral quantification

Viral loads in NP swabs were assessed based on standard curves generated by amplification of the matrix gene targets for influenza A and B using conventional RT-PCR, cloning of each target into plasmids and performance of real-time RT-PCR on serial dilutions of the quantified plasmids.

### Analytical sensitivity

To directly compare the limits of detection (LoD) of RV15 and the WHO real-time RT-PCRs, parallel testing was performed using 10-fold serially diluted viruses. The viruses used were reference strains of the CIRN SOS Network that had been characterized by the National Microbiology Laboratory (Winnipeg, MB) as [A/California/7/2009 (H1N1)], [A/Perth/16/2009 (H3N2)], [B/Brisbane/60/2008 (Victoria lineage)] and [B/Wisconsin/1/2010 (Yamagata lineage)]. Triplicates values from three independent experiments were analysed, and the *C*_t_ values obtained from real-time RT-PCR for influenza A and B were used to estimate viral concentrations (relative to the standard curves generated using plasmid controls). The LoD was estimated at a probability of 95 % by Probit analysis [36, 41] using StatPlus 2009 Professional version 5.7.8.

## Results

### Number of influenza A and B cases evaluated

**Fig. 1.**
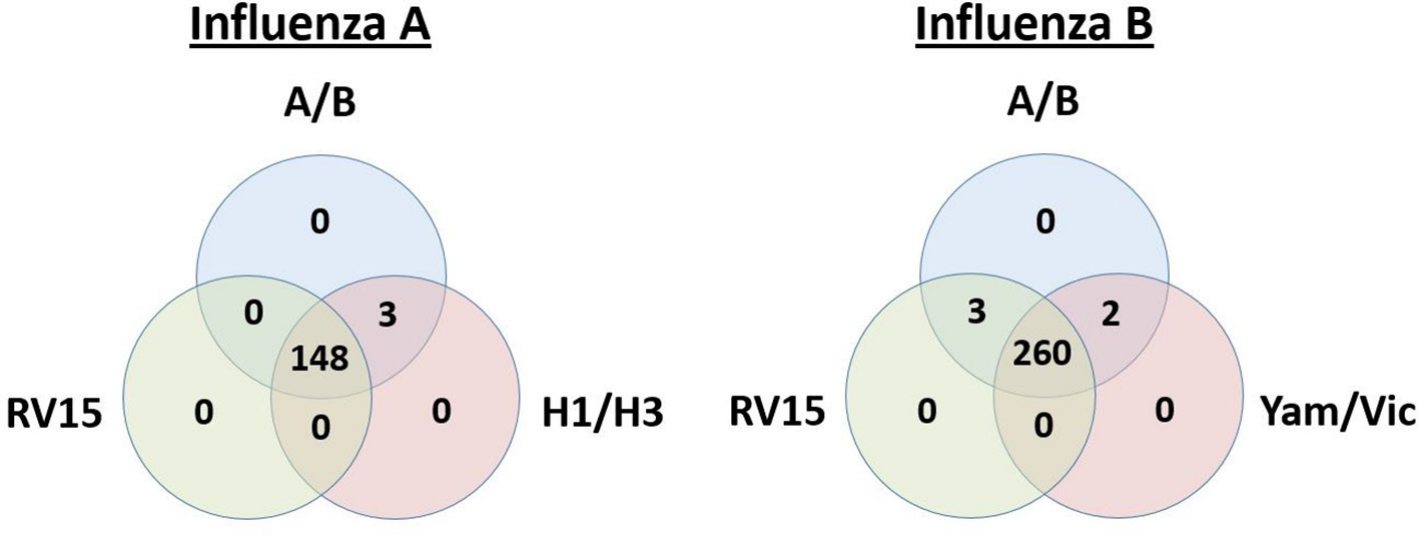
Overlap of positive influenza A or B results between RV15 and the WHO real-time RT-PCRs. Venn diagrams illustrating the overlap of positive results for influenza A or influenza B in patients tested by the Seegene multiplex PCR (RV15), the WHO real-time RT-PCRs for influenza A or B detection (A/B) and influenza A subtyping (H1/H3) or influenza B discrimination for Yamagata or Victoria (Yam/Vic) lineages.

**Fig. 2.**
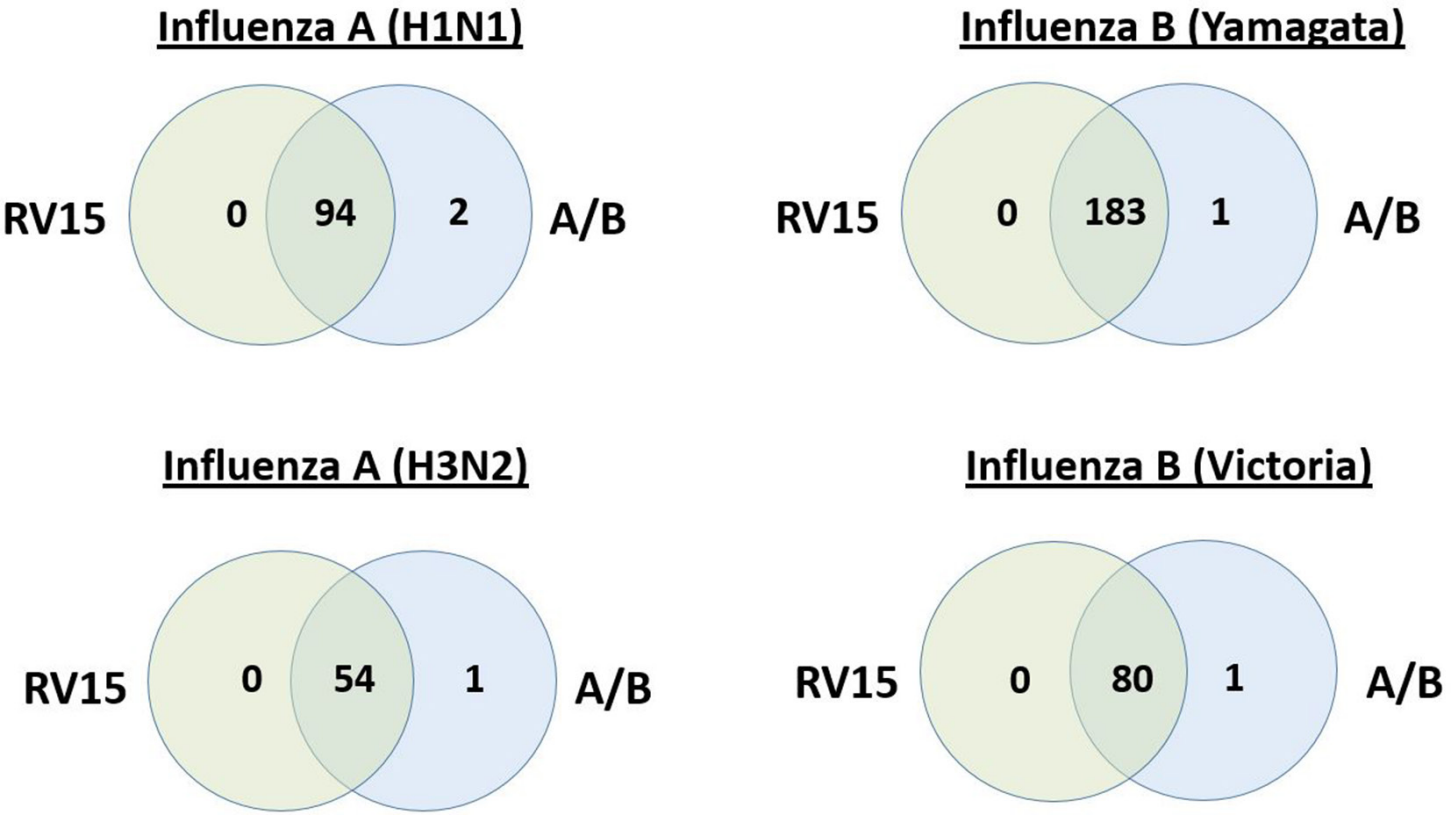
Overlap of results between RV15 and the WHO real-time RT-PCRs for characterized influenza A or B viruses. Venn diagram illustrating the overlap of positive results between RV15 and influenza A/B real-time RT-PCRs for characterized influenza A subtypes (H1N1 or H3N2) and influenza B lineages (Yamagata or Victoria).

Laboratory data were available on 1111 adults hospitalized with acute respiratory illness, who had all 3 molecular tests of interest performed: RV15, influenza A/B duplex real-time RT-PCR for influenza A or B screening; and H1/H3 real-time RT-PCR subtyping for positive influenza A specimens or real-time RT-PCR Yamagata/Victoria lineage discrimination for positive influenza B. Of the 1111 patients, 151 were positive for influenza A (96 H1N1 and 55 H3N2) and 265 were positive for influenza B (184 Yamagata lineage and 81 Victoria lineage). Most of the results overlapped between testing methods, but some discrepant results were observed (Figs 1 and 2).

### Performance of RV15 for detection of influenza A and B

Compared to the composite reference standard, RV15 showed a sensitivity for influenza A of 98.0 % (148/151; 95 % CI: 95.8–98.0 %) and a specificity of 100.0 % (960/960; 95 % CI: 99.6–100.0 %). Discordant results were seen in three influenza A positive results (Table 1; Fig. 1). The accuracy of RV15 for influenza A was 99.7 % (1108/1111; 95 % CI: 99.1–99.7 %). Similar performance characteristics were noted for influenza A H1N1 and H3N2 (Table 1; Fig. 2). For influenza B, RV15 showed a sensitivity of 99.2 % (263/265; 95 % CI: 98.0–99.2 %) and a specificity of 100.0 % (846/846; 95 % CI: 99.6–100.0 %). Discordant results were seen in three influenza B positive results (Table 1; Fig. 1). The accuracy of RV15 for influenza B was 99.8 % (1109/1111; 95 % CI: 99.2–99.8 %). Similar performance characteristics were noted for the influenza B Yamagata and Victoria lineages (Table 1; Fig. 2). Overall, no significant differences were noted between real-time RT-PCR methods or RV15 and the composite reference standard, for all viruses, influenza A subtypes or influenza B lineages (with *P* values ranging from 0.248 to 1.000).

**Table 1.** Assay performance against the composite reference standard

Assay	Target	Performance characteristics								
		TP	TN	FP	FN	Sensitivity (95 % CI)	Specificity (95 % CI)	Accuracy (95 % CI)	Misclassification rate (95 % CI)	Kappa (95 % CI)
FluA/B real-time RT-PCR	FluA (total)	151	960	0	0	100.0 (98.1–100.0)	100.0 (99.7–100.0)	100.0 (99.5–100.0)	0.0 (0.0–0.5)	100.0 (97.8–100.0)
	FluA (H1N1)	96	1015	0	0	100.0 (97.1–100.0)	100.0 (99.7–100.0)	100.0 (99.5–100.0)	0.0 (0.0–0.5)	100.0 (96.8–100.0)
	FluA (H3N2)	55	1056	0	0	100.0 (94.9–100.0)	100.0 (99.7–100.0)	100.0 (99.5–100.0)	0.0 (0.0–0.5)	100.0 (94.7–100.0)
	FluB (Total)	265	846	0	0	100.0 (98.9–100.0)	100.0 (99.7–100.0)	100.0 (99.5–100.0)	0.0 (0.0–0.5)	100.0 (98.6–100.0)
	FluB (Yamagata)	184	927	0	0	100.0 (98.5–100.0)	100.0 (99.7–100.0)	100.0 (99.5–100.0)	0.0 (0.0–0.5)	100.0 (98.2–100.0)
	FluB (Victoria)	81	1030	0	0	100.0 (96.5–100.0)	100.0 (99.7–100.0)	100.0 (99.5–100.0)	0.0 (0.0–0.5)	96.3 (96.3–100.0)
RV15	FluA (total)	148	960	0	3	98.0 (95.8–98.0)	100.0 (99.6–100.0)	99.7 (99.1–99.7)	0.3 (0.3–0.9)	98.8 (96.2–98.8)
	FluA (H1N1)	94	1015	0	2	97.9 (94.9–97.9)	100.0 (99.7–100.0)	99.8 (99.2–99.8)	0.2 (0.2–0.8)	98.8 (95.1–98.8)
	FluA (H3N2)	54	1056	0	1	98.2 (92.7–98.2)	100.0 (99.7–100.0)	99.9 (99.4–99.9)	0.1 (0.1–0.6)	99.0 (93.2–99.0)
	FluB (Total)	263	846	0	2	99.2 (98.0–99.2)	100.0 (99.6–100.0)	99.8 (99.2–99.8)	0.2 (0.2–0.8)	99.5 (97.9–99.5)
	FluB (Yamagata)	183	927	0	1	99.5 (97.8–99.5)	100.0 (99.7–100.0)	99.9 (99.4–99.9)	0.1 (0.1–0.6)	99.7 (97.7–99.7)
	FluB (Victoria)	80	1030	0	1	98.8 (95.0–98.8)	100.0 (99.7–100.0)	99.9 (99.4–99.9)	0.1 (0.2–0.6)	99.3 (95.2–99.3)

CIs, confidence intervals; FluA, influenza A; FluB, influenza B; FN, false negative; FP, false positive; TN, true negative; TP, true positive.

### Discrepant analyses

For influenza A specimens not detected by RV15, the *C*_t_ values obtained with influenza A/B real-time RT-PCR were 32.3, 34.2 and 34.5, which is near the previously established detection limit (i.e. *C*_t_ cutoff of 36.4) (Table S2). Similarly, the *C*_t_ values for influenza B real-time RT-PCR results in cases not detected by RV15 were 34.3 and 36.9, where the previously established *C*_t_ cutoff for influenza B was 37.3 (Table S2). These results are consistent with specimens with low viral loads, spanning concentrations of 1.7, 2.0, and 4.9 copies/reaction for influenza A and 0.2 and 0.8 copies/reaction for influenza B. Upon retest of discrepant results by RV15, all results remained negative, whereas testing at the National Microbiology Microbiology (Winnipeg, MB) confirmed the positive results by RT-PCR and sequencing.

### Analytical sensitivity

In this study, the estimated LoD of RV15 for each influenza A subtype and influenza B lineage was higher than that of the real-time RT-PCR for influenza A/B (Table 2), but below the detection limits of the real-time RT-PCRs for influenza A subtyping or B lineage characterization (Tables 2 and S3). This is consistent with previously validated cutoffs for positivity for the real-time RT-PCR methods (Table S2). At viral concentrations that were below the detectable limit of RV15 but detected using influenza A/B real-time RT-PCR, the *C*_t_ values from the real-time RT-PCR spanned from 32.7 to 34.1 for influenza A H1N1, from 32.0 to 34.6 for influenza A H3N2, from 36.1 to 37.3 for influenza B Yamagata lineage and from 34.1 to 36.8 for influenza B Victoria lineage (data not shown). These *C*_t_ values represent concentrations near the assay cutoff values for each target (Table 2 and S2).

**Table 2.** Estimated LoD for detection of influenza A subtype or influenza B lineages

Virus	LoD in copies/reaction* (95 % CI)			
	RV15	Real-time RT-PCR influenza A/B detection	Real-time RT-PCR influenza A H1/H3 subtyping	Real-time RT-PCR influenza B Yamagata/Victoria lineage
FluA H1N1	4.0 (3.2–6.8)	1.5 (1.2–2.5)	9.5 (7.0–12.2)	na
FluA H3N2	4.2 (2.2–5.6)	0.9 (0.7–1.3)	0.9 (0.7–1.3)	na
FluB Yamagata	5.2 (4.0–6.4)	0.4 (0.1–0.6)	na	25.0 (22.6–26.6)
FluB Victoria	3.2 (3.0–3.4)	1.5 (1.1–1.4)	na	3.2 (2.6–3.9)

*The concentration that could reproducibly (at 95 % confidence) be detected by the test method is described as the LoD. The LoD was determined by Probit analysis by testing replicate aliquots of virus dilutions (see Table S3). For each virus aliquot, the *C*_t_ values obtained by the WHO real-time RT-PCR were used to infer viral load. Concentrations of virus dilutions were estimated using standard curves generated with plasmids pFluA and pFluB from this study (see Supplementary Material).

CI, confidence intervals; FluA, influenza A; FluB, influenza B; LoD, limit of detection; na, not applicable.

## Discussion

The CIRN SOS Network uses WHO-based real-time RT-PCRs to identify and characterize influenza A subtypes and influenza B lineages. Compared to the WHO reference methods, the RV15 assay had an accuracy of 99.7 and 99.8 % for the detection of influenza A and B, respectively.

While this was not statistically significant, RV15 was unable to detect influenza A or B in a small subset of specimens in which the viral loads were low. Conventional multiplex PCRs such as RV15 are often less sensitive than real-time RT-PCR, and this can be explained in part by the assay principle. Real-time RT-PCR detects fluorescence signals captured during PCR amplification cycles, and results are defined objectively using validated cutoffs for positivity. Conventional RT-PCR is an end-point detection of amplicons following electrophoresis and staining; a process where visualization of amplicons can be difficult and subjective when working with specimens with low concentrations of target [15–17, 21–23, 36, 41].

Whether conventional or real-time RT-PCR is used, the performance of these molecular methods can also vary, depending on factors such as genetic mismatches in the PCR target region, which could arise in influenza over time via antigenic drift. This emphasizes the need to verify the performance for characterized and circulating subtypes and lineages of influenza. However, for RV15, only a limited number of studies have assessed its ability to detect influenza A and B viruses, and none have specifically looked at its performance for the detection of influenza A virus subtypes or influenza B lineages [15, 19, 20, 23, 35]. Cho *et al*. [23] compared RV15 to a composite reference standard that included culture and a commercial real-time RT-PCR and demonstrated that RV15 had a sensitivity of 93.4 % (with 95 % CI: 88.8–93.4 %) for influenza A and one of 79.5 % (95 % CI: 79.0–88.6 %) for influenza B. Gharabaghi *et al*. [19] assessed the performance of RV15 against direct fluorescent antibody testing, virus culture and isolation, and three additional multiplex PCR methods. The specificities for influenza A and B were 98.8 and 100 %, and the sensitivities were 96.9 % (95 % CI: 91.9–96.9 %) and 100 % (92.6–100 %), respectively. In the present study, the performance of RV15 was verified for the detection of recent influenza A subtypes (H1N1 and H3N2) and influenza B lineages (Yamagata and Victoria), and it showed similar performance characteristics to the WHO real-time RT-PCR. Since this study used specimen collected prospectively from 2011 to 2013, it could be argued that the performance of the molecular methods assessed could vary with more recent circulating influenza strains, if primer or probe mismatches occur in the assay gene targets. However, the CIRN SOS laboratory participates in yearly quality assurance programmes proficiency testing for influenza A and B detection and characterization using all the assays from this study (all real-time RT-PCRs and the RV15 assay), and no differences in assay performance have been observed over time (data not shown). In this study, only a small subset of results in specimens with low viral loads were not detected by RV15, with this representing 0.3 and 0.2 % of the total tests for influenza A and B, respectively. While the lower sensitivity of multiplex PCRs such as RV15 was expected, the possibility of target gene sequence mismatches cannot be excluded.

It should be noted that viral loads were estimated using quantification relative to a standard curve generated using plasmid DNA controls. With influenza virus being an RNA virus, comparison to plasmid DNA does not account for the reverse transcription step. Regardless, all viruses were subjected to direct method comparison in the analytical analyses, which includes the reverse transcription step. Subsequently, the resulting *C*_t_ values were subjected to comparison against the standard curve derived from plasmid DNA results, as plasmid DNA was more readily quantifiable. Overall, with this approach it remains valid to infer that the specimens not detected by RV15 had low viral loads, but the absolute quantity of virus should be considered to be an estimate (Tables 2, S2 and S3). Overall, this would have little impact on the conclusions from this study.

This study’s strengths include prospectively collected specimens from a defined patient population (adults hospitalized with acute respiratory illness), comparison of results against reference methods for influenza A and B detection, and analyses performed on influenza viruses characterized by subtyping or lineage determination. The main limitation of the study was that it was focused solely on influenza virus. However, this is justified because influenza viruses are the only respiratory viruses for which vaccines are currently available, and the performance for the detection of other viruses has been assessed previously [17, 19–23]. While assessing the performance of RV15 against validated real-time RT-PCRs for influenza is well justified, this study focused on the use of RV15 for population-based surveillance in hospitalized adults. These findings should not be extrapolated to other patient populations or for applications in clinical diagnostic testing where individual-level results are prioritized. The patients tested in this study were hospitalized adults, often presenting with co-morbidities and severe outcomes [1–7]. Further, respiratory virus testing is often performed using testing algorithms following initial screening methods for influenza A and B. As such, nucleic acids extracted from clinical specimens are sometimes inadvertently subjected to a freeze/thaw cycle prior to testing. The impact of freeze/thaw was not assessed in this study, as testing was performed following independent nucleic acid extraction on a different specimen aliquot. However, given that RV15 failed to detect a small subset of influenza A and B specimens at low viral loads, additional freeze/thaw cycles may further compromise influenza virus detection, and should thus be avoided. Finally, the workflow is relatively simple with RV15 for small numbers of specimens, but additional benefits could be afforded by using imaging software enabling automated amplicon detection if high-throughput specimen processing is required [15–17, 19–23]. Such automated analyses of RV15 amplicons could also reduce reduce result subjectivity compared to interpretations made from visual assessment of amplicons [15–17, 19–23]. Automated analyses were not evaluated in this study, but the technical staff performing RV15 testing were blinded to the real-time RT-PCR method results to avoid bias, and the RV15 results were remained unchanged with subsequent independent review by other blinded staff members.

Overall, the performance of RV15 was comparable to the WHO real-time RT-PCR standards for the detection of recently circulating subtypes of influenza A and lineages of influenza B, and it only missed a very small subset of influenza A and B results at low viral loads. Given that the performance characteristics of the RV15 multiplex PCR are not provided in the manufacturer kit insert, these data are of value for its users, which include several acute care hospitals and provincial public health laboratories in Canada [36, 41]. This study shows that the RV15 conventional multiplex PCR can be used for surveillance studies for respiratory viruses without significantly compromising detection of influenza A and B. The use of multiplex technologies such as RV15 can help better define the epidemiology of influenza and NIRVs, and these data are important for the development of novel therapeutics and vaccines. 

## Supplementary Data

Supplementary material 1
