# Supplementary material for: Validation of the Seegene RV15 multiplex PCR for the detection of influenza A subtypes and influenza B lineages during national influenza surveillance in hospitalized adults
Source: J Med Microbiol. 2019 Jul 2;69(2):256–64. doi: 10.1099/jmm.0.001032 (PMC7431100; doi:10.1099/jmm.0.001032)
Supplement: Supplementary material 1 [file jmm-69-256-s001.pdf]

Table S1. Oligonucleotides used in this study

Name	Sequence (5'->3')	Reference
InfA-F	GACCRATCCTGTCACCTCTGAC	12
InfA-R	AGGGCATTYTGGACAAAKCGTCTA	12
InfA-Pr[1]	FAM-TGCAGTCCTCGCTCACTGGGCACG-BHQ1	12
InfB-F	GAGACACAATTGCCTACCTGCTT	13
InfB-R	TTCTTTCCCACCGAACCAAC	13
InfB-Pr[1]	HEX-AGAAGATGGAGAAGGCAAAGCAGAACTAGC-BHQ1	13
SwH1p-F	GTGCTATAAACACCAGCCTYCCA	12
SwH1p-R	CGGGATATTCCTTAATCCTGTRGC	12
SwH1p-Pr[1]	FAM-CAGAATATACAT[BHQ1]CCRGTCACAATTGGARAA-SpC3	12
A/H3s-F	GATGTGTACAGAGATGAAGCATTAAACA	13
A/H3s-R	TAGGATCCAATCTTTGTATCCTGACTT	13
A/H3s-Pr1/2[2]	HEX-AGCTCAACRCCTTTGATCTGGAACCGG-3IABkFQ	13
InfB/HA-F (F432)	ACCCTACARAMTTGGAACYTCAGG	14
InfB/HA-R (R479)	ACAGCCCAAGCCATTGTTG	14
InfB/HA-Yam Pr (MGB437)[3]	FAM-AATCCGMTYTTACTGGTAG-MGB	14
InfB/HA-Vic Pr (MGB437)[3]	VIC-ATCCGTTTCCATTGGTAA-MGB	14
FASphI	TGC<u>GCATGC</u>GACCRATCCTGTCACCTCTGAC	This study
RASacI	GGC<u>GAGCTC</u>AGGGCATTYTGGACAAAKCGTCTA	This study
FBSacI	GGC<u>GAGCTC</u>GAGACACAATTGCCTACCTGCTT	This study
RBSphI	TGC<u>GCATGC</u>TTCTTTCCCACCGAACCAAC	This study

All primers were synthesized by Sigma Genosys. Probes were synthesized from different sources, and were either BioSearch Technologies[1], Integrated DNA Technologies[2], or Applied Biosystems[3] (Life Technologies).

Table S2: Previously established positivity cutoffs for the real-time RT-PCRs used in this study.

Virus	Real-time RT-PCR method			
	Influenza A/B detection		Influenza A H1/H3 subtyping	Influenza B Yamagata/Victoria lineage characterization
	Estimated virus concentration in copies/reaction (95% CI)*	Ct cutoff**	Ct cutoff**	Ct cutoff**
FluA H1N1	0.9 (0.3-3.5)	35.7	38.2	N/A
FluA H3N2	2.6 (1.0-6.8)	36.5	39.3	N/A
FluA (total)	0.4 (0.1-1.4)	36.4	N/A	N/A
FluB Yamagata	0.1 (0.01-1.0)	38.1	N/A	36.2
FluB Victoria	0.04 (0.01-5.8)	36.5	N/A	38.8
FluB (total)	0.01 (0.1-0.003)	37.3	N/A	N/A

*Cutoff values were determined at values where 95% of positive results were detected for n = 24 (10-fold serial dilutions of each virus, tested in triplicate by two operators on four separate days). The WHO real-time RT-PCR methods were used to test virus dilution, and Ct values were determined by the manufacturer software, using validated thresholds to define cutoffs for positivity. The threshold values were set at 2.2×10^4 for influenza A/ real-time RT-PCR and influenza A H1/H3 subtyping, at 1.4×10^5 for influenza B Yamagata A and 5.1×10^4 for influenza B Victoria.

**Concentrations of virus dilutions were estimated using standard curves generated with plasmids pFluA and pFluB from this study (see Supplementary Material). Abbreviations: Confidence intervals (CI); influenza A (FluA); influenza B (FluB); limit of detection (LoD); not applicable (N/A); standard deviation.

Note: The validation data for the WHO real-time RT-PCRs by the CIRN SOS Network was presented at the IDWeek Meeting of the Infectious Diseases Society of America (IDSA), San Francisco, October 3, 2013. The corresponding publication "ElSherif et al. 2013. Validation of real-time reverse transcriptase polymerase chain reaction (rRT-PCR) assays for the detection and characterization of influenza strains. Poster 281" is freely accessible at the following

website: https://idsa.confex.com/idsa/2013/webprogram/Handout/id1838/POSTER49_281.pdf) [last accessed January 3, 2019].

Table S3: Probability of detection of influenza A and B at low viral loads.

Virus	Estimated virus concentration in copies/reaction (95% CI)*	Probability of detection (%)**			
		RV15	Real-time RT-PCR influenza A/B detection	Real-time RT-PCR influenza A H1/H3 subtyping	Real-time RT-PCR influenza B Yamagata/Victoria lineage
Influenza A subtype H1N1	94.7 (76.8-117.8)	100	100	100	N/A
	12.8 (9.7-16.2)	100	100	100	N/A
	2.6 (2.0-4.0)	100	100	0	N/A
	0.7 (0.5-0.9)	0	66.67	0	N/A
	0.3 (0.2-0.4)	0	0	0	N/A
Influenza A subtype H3N2	58.6 (56.6-62.6)	100	100	100	N/A
	6.9 (5.6-7.8)	100	100	100	N/A
	1.2 (0.8-1.6)	66.67	100	100	N/A
	0.1 (0.0-0.2)	0	0	0	N/A
Influenza B Yamagata lineage	27.7 (25.3-29.6)	100	100	N/A	100
	5 (4.3-5.8)	100	100	N/A	55.6
	0.6 (0.1-0.8)	66.67	100	N/A	0
	0.1 (0.0-0.2)	0	0	N/A	0
Influenza B Victoria lineage	81.0 (60.3-130.2)	100	100	N/A	100
	9.6 (7.3-14.3)	100	100	N/A	100
	2.0 (1.8-2.2)	100	100	N/A	66.67
	0.5 (0.4-0.6)	0	33.3	N/A	0
	0.1 (0.0-0.2)	0	0	N/A	0

* For each virus dilution, the Ct values obtained by the WHO real-time RT-PCR were used to infer viral load. Concentrations of virus dilutions were estimated using standard curves generated with plasmids pFluA and pFluB from this study (see Supplementary Material).

**Probability of detection of nucleic acids derived from virus dilutions, based on three independent dilutions series tested in triplicate (n=9).

Supplementary Material - Document S1: Methods used for the plasmid control for quantification of influenza A and B

Conventional RT-PCR for molecular cloning

A Qiagen OneStep RT-PCR Kit was used for conventional RT-PCR in 50 µl reactions consisting of: 2 µl enzyme mix; 1× buffer; 400 µM dNTPs, 1 µM of forward and reverse primer pairs for influenza A (FASphI and RASacI) or influenza B (FBSacI and RBSphI) (Table S1); and 5 µl template TNA extracted from influenza A or B virus. Amplifications were performed using a C1000 Touch thermocycler (BioRad Laboratories Inc.) as follows: 50°C for 30 min; 94°C for 5 min; and 35 cycles of 50°C for 30 s, 72°C for 1 min, and 94°C for 1 min. The resulting 123 and 113 base pairs (bp) for influenza A and B amplicons were resolved using 1% agarose gel electrophoresis with ethidium bromide staining, as described for the RV15 respiratory virus multiplex PCR. Amplicon DNA was purified using a QIAquick Gel Extraction Kit (Qiagen Inc., Mississauga, ON) following manufacturer's recommendations.

Molecular cloning

Amplicon and plasmid pGEM-T Easy (Promega), were subjected to SacI-HF and SphI-HF double restriction endonuclease digestion in 1× CutSmart buffer, as per manufacturer instructions (New England Biolabs, Inc.). Following overnight incubation at 37°C, and purification, the amplicons were ligated into the purified digested plasmid in 25 µl reactions containing 800 units of T4 DNA ligase (New England Biolabs Inc.), 1× T4 DNA ligase buffer, and equivalent volumes of the amplicon and plasmid DNA. After 30 minutes at 14°C, the ligation mixture was electroporated into electrocompetent *Escherichia coli* TOP10 (ThermoFisher Scientific) using a Gene Pulser (BioRad Laboratories) at 2.1 kV, 200 Ω and 25 µFD. Transformants were selected on Luria-Bertani (LB) agar with 100 µg/ml of ampicillin. Individual colonies were subjected to TNA extraction and real-time RT-PCR for influenza A/B, as previously described. Positive colonies we inoculated into LB broth with selection, and plasmids were purified using a QIAprep Spin Miniprep Kit (Qiagen Inc),

as recommended by the manufacturer. Plasmids containing the influenza A or B matrix genes were termed pFluA and pFluB, respectively.

Plasmid quantification and generation of influenza A and B standard curves

Each plasmid was quantified using UV spectrophotometry using a NanoVue Plus instrument (GE Healthcare, Mississauga ON). Genome equivalents were estimated using the amount (g) of target, the average weight of a base pair (650 g/mol), Avogadro's number (6.022×10^{23} molecules/mol), using the following formula: [gene amount (g)/length of the target (bp) × 650 (g/mol)] × (6.022×10^{23} molecules/mol). Plasmids pFluA and pFluB were subjected to three independent 10-fold serial dilutions, and each dilution was tested in triplicate using real-time RT-PCR for influenza A/B. The Ct values were plotted against the $\log_{10}$ value of the virus concentration, and expressed as log copies/reaction +/- standard deviation (SD).
